# The Microbiome Modulates the Immune System to Influence Cancer Therapy

**DOI:** 10.3390/cancers16040779

**Published:** 2024-02-14

**Authors:** Ruchi Roy, Sunil Kumar Singh

**Affiliations:** 1 UICentre for Drug Discovery, The University of Illinois, Chicago, IL 60612, USA; 2 Department of Surgery, Division of Surgical Oncology, The University of Illinois at Chicago, Chicago, IL 60612, USA

**Keywords:** probiotics, microbiota, cancer immune therapy

## Abstract

**Simple Summary:**

This review discussed the importance of the gut microbiome for cancer patients and how disturbances in the microbiome can lead to unwanted side effects during cancer treatment. The way in which a variety of foods can influence the microbiota and its abundance was described. Not only this, the effects of antibiotics can also modulate the abundance of the gut microbiome and its diversity. Alterations in the bacterial population can help achieve better immune responses and maintain the intestinal barrier’s integrity. In this regard, this article also discussed several advanced approaches utilizing the advantages of probiotics along with immunotherapies.

**Abstract:**

The gut microbiota composition can affect the tumor microenvironment and its interaction with the immune system, thereby having implications for treatment predictions. This article reviews the studies available to better understand how the gut microbiome helps the immune system fight cancer. To describe this fact, different mechanisms and approaches utilizing probiotics to improve advancements in cancer treatment will be discussed. Moreover, not only calorie intake but also the variety and quality of diet can influence cancer patients’ immunotherapy treatment because dietary patterns can impair immunological activities either by stimulating or suppressing innate and adaptive immunity. Therefore, it is interesting and critical to understand gut microbiome composition as a biomarker to predict cancer immunotherapy outcomes and responses. Here, more emphasis will be given to the recent development in immunotherapies utilizing microbiota to improve cancer therapies, which is beneficial for cancer patients.

## 1. Introduction

Cancer is the second leading cause of death worldwide according to the World Health Organization (WHO), affecting over 9 million people worldwide [1]. Epidemiological studies have shown that diet is crucial for influencing the risk of cancer. In 2018, the WHO emphasized the association between diet and lifestyle-related risk factors and the burden of diseases [2]. Evidence supports that several cancers’ development and treatment can be affected by modifying diet and nutrition [3]. However, the association between diet and cancer cannot be completely derived, sometimes due to inherent biases and some other factors. These factors include environmental stress, eating habits, alcohol, obesity, inappropriate physical activity, high blood glucose, cholesterol level, and fruit and vegetable intake, which accounted for 18% and 25% of the world’s death burden in low- and middle-income countries and high-income countries, respectively [4,5]. Therefore, studies are more focused on restricting diet and energy intake to influence cancer development and its treatment [6]. Here, we describe the quality of diet that can influence cancer treatment and the biomarkers associated with digestive tract cancers. Although there are therapeutic strategies available to treat cancer patients, they do not completely enhance their lifestyles and can cause them to suffer associated side effects. These treatments greatly impact their day-to-day lives due to the pain associated with them, particularly chemotherapy and radiotherapy, which cause severe abdominal pain, diarrhea, mucositis, vomiting, constipation, etc. [7]. These therapies alter the composition of the intestinal microbiota, i.e., dysbiosis, leading to immunological dysregulation and sometimes resistance to some bacteria. For example, paclitaxel, a known chemotherapeutic agent, not only affects the gut microbiome, colonic tissue integrity, and microglia activation but also increases some pro-inflammatory agents like matrix metalloproteinase 9 (MMP9) and tumor necrosis factor-alpha (TNF-α) that alter bacterial diversity in the colon [8]. Nonetheless, gut microbiota may accelerate the development of cancer in several ways, such as by causing DNA damage or genetic alteration, affecting cell cycle progression through its products, making pro-tumorigenic microbial niches, including biofilms, or creating immune dysregulation.

**Figure 1:**
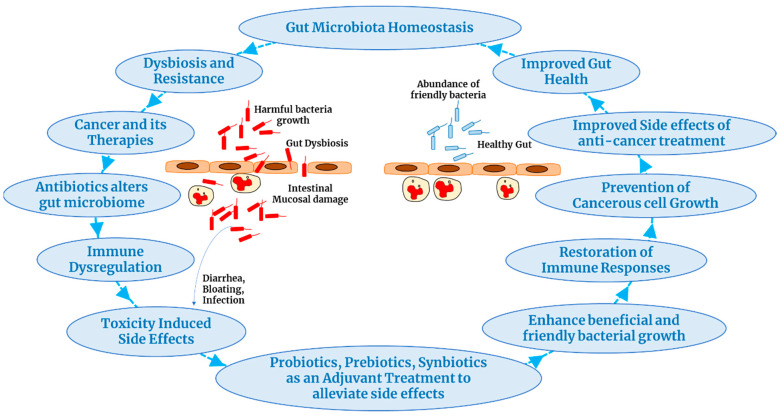
Manipulating the gut microbiota to alleviate cancer therapies’ side effects.

Thus, in this article, we discuss how any diseased condition or even cancer development can make changes to the gut microbiota and its influence on immune responses. Moreover, we address how the side effects or symptoms associated with cancer treatment can be cured or alleviated with the administration of probiotics, which can also help or facilitate cancer treatment (Figure 1). In addition, we discuss in detail how various treatment procedures involving the usage of live microorganisms (probiotics) or dietary fiber fermented by intestinal microorganisms (prebiotics) are being studied and used to help the growth and abundance of friendly bacteria modify the gut microbiota, preventing dysbiosis and maintaining their homeostasis condition to control cancer progression.

## 2. Poor Quality of Food Increases the Risk of Cancer

In 2018, the World Cancer Research Fund reported an inconclusive association between fruits or vegetables in the diet and the risk of any cancer [2]. Usually, the risk of cancer in vegetarians is less compared to nonvegetarians because their diet is more dependent on fruit and vegetables than nonvegetarians, but this cannot be true for each and every individual cancer type [9]. It is evident that the long-term health of vegetarians is comparatively better than that of nonvegetarians due to a lower prevalence of health issues such as weight gain, obesity, diabetes, stroke, and cancer. The intake of processed meat and cheeses could be one of the reasons for increasing cancer risk because of their high content of nitrite/nitrate [10,11]. Also, the International Agency for Research on Cancer (IARC) in 2018 documented the toxic effects of processed or unprocessed meat due to the presence of haem iron, which leads to an enhanced formation of nitroso compounds in the gastrointestinal tract, e.g., S-nitrosothiols and the nitrosyl heme [12,13]. These nitroso compounds can alkylate guanine to form the promutagenic DNA lesions O^6^-methylguanine and O^6^-carboxymethylguanine. This mutation can cause cancer if not repaired. Moreover, the cooking of meat at high temperatures can produce mutagenic heterocyclic amines and polycyclic aromatic hydrocarbons, which can also be one of the reasons that increase the risk of cancer [14].

A detailed study by Reynolds in 2019 explained the association between carbohydrate quality and human health by performing extensive meta-analyses of prospective studies and reviewing systematic published reviews from databases. Their findings, based on a thorough search of the literature available in databases such as PubMed, Embase, Ovid MEDLINE, and the Cochrane Central Register of Controlled Trials, suggested that higher intakes of whole grains were associated with a 15 to 30% reduction in the risk for all critical outcomes, including cardiovascular-associated mortality, colorectal cancer, and type 2 diabetes [15]. High dietary folate [16] and high dietary fiber consumption can reduce the rate of colorectal cancer. Especially cereal fiber and wholegrain cereals are more protective than fiber from fruit or vegetables [15,17]. However, dietary fibers are controversial and classified as probable causes of cancer as per the World Cancer Research Fund (WCRF) and the American Institute for Cancer Research (AICR) [10]. Reports from the WCRF and the AICR provide updates on specific cancers and guidance for cancer patients and survivors on diet and lifestyle changes. These reports also benefit individuals who are at risk due to genetic predisposition or carcinogenic exposures [10]. Among the most probable causes associated with cancer risk is alcohol consumption, as it can lead to the development of cirrhosis and alcoholic hepatitis. The second most probable cause of cancer is overweight and obesity [18]. An association between vegetable intake and cancer risk has been established among women but not observed among men. A detailed study, including several parameters such as age, sex, smoking, energy intake, ethnicity, and other potential dietary factors, revealed a higher intake of nonstarchy vegetables, salt, pickled food, and processed meat is inversely associated with the risk of bladder and gastric cancer [19,20,21].

Foods containing vitamin C, calcium supplements [22], and folate supplements help to suppress the development of tumors in normal tissues [23]. Folate is required for DNA synthesis and replication, and its deficiency can result in ineffective DNA synthesis and the transformation of normal cells into neoplastic ones. Despite these components being added as a supplement factor in fortified food as a protective factor that may decrease the risk of prostate cancer, folic acid is listed as a nutrient that may induce prostate cancer risk. However, there are many controversial reports on the association of the intake of folate with cancer; for example, studies have not reached a sufficient significance level to comment on its direct associations in the case of prostate cancer [24,25]. A dose–response meta-analysis-based study by Wang et al. summarized the evidence regarding the associations of folate intake and serum folate levels with prostate cancer risk [25]. They found no significant effect of dietary folate intake on prostate risk; however, increased serum folate levels can be associated with the risk of prostate cancer. In addition, metabolomics studies by Liss et al. identified significantly altered folate and arginine pathways in the gut microbiome, which suggests that gut bacteria might affect the risk of prostate cancer [26]. Therefore, it is difficult to conclude the importance of folate in prostate cancer.

Similarly, the consumption of coffee reduces cancer risk due to the presence of strong antioxidants and anti-inflammatory agents, such as polyphenolic compounds. A bibliographic study by Pauwels et al., 2021, based on a search on PubMed and Embase, identified that intake of coffee is inversely associated with the risk of hepatocellular cancer and breast cancer among postmenopausal women [21]. The reason for this association and the beneficiary effect of coffee was ascribed to the presence of some bioactive components such as caffeine, cafestol, kahweol, and chlorogenic acids. The protective role of coffee was investigated and confirmed by another population-based case–control study, where coffee was proposed as a protective factor for colorectal cancer because it reduces the cancer risk by 26% [27]. However, this search could not comment clearly on this association with other cancer types, including esophagus, pancreas, colorectum, kidneys, bladder, ovaries, and prostate, because the results were inconclusive or the data were insignificant in the reports found. Thus, straightforward evidence is needed before recommending any food or beverage consumption.

A Mediterranean-style dietary pattern (MSDP) also strongly lowers the risk of cancer, as shown in a semi-quantitative food frequency questionnaire-based exam involving 2966 participants of the Framingham Offspring who were free of prevalent cancer. This study showed the beneficial effects of an MSDP on cancer risk. In this study, MSDPs had a beneficial effect on women, showing a lower risk of cancer as compared to men, who have higher adherence to MSDPs [28].

The role of sugar and salt is important for the taste of food, but if they are taken in a disproportionate amount, they can also pose a risk to health. A study by Wu et al., 2021, performed a systematic review and meta-analysis to determine the association of salt intake with gastric cancer morbidity and mortality. This cohort study observed that highly but not moderately pickled food intake increases gastric cancer risk, whereas no effect of moderate or high salted fish intake was associated with gastric cancer risk. Moreover, it is advisable to not consume fermented Cantonese-style salted fish or to have a high intake of processed meat, as they can be associated with a higher risk of gastric cancer [29,30]. Another study by Strumylaite et al., 2006, reported that the consumption of salted mushrooms might increase the risk of gastric cancer for people who like salty food, salt-preserved meat, and fish [31].

Contaminated food with certain fungi that are found on crops may also increase the risk of liver cancer. Toxins produced by *Aspergillus flavus* and *Aspergillus parasiticus*, i.e., aflatoxins, which are abundant in warm and humid regions of the world, are known to be associated with an increased risk of liver cancer [32]. Not only can contaminated food be the cause of an increased risk of cancer, but drinking water contaminated with arsenic can also cause solid cancers of the lungs, urinary bladder, skin, kidneys, liver, and prostate. Arsenic is a known carcinogen that can enter the body through exposure to arsenic-contaminated drinking water. This was shown in a recent cohort study by Issanov et al., 2023, and Lin et al., 2022, where they highlighted the relationship between arsenic in drinking water and the incidences of lymphoma and leukemia by sex, exposure category, and time period [33,34]. These studies suggested that exposure to arsenic increases the risk of developing bladder and kidney cancers.

## 3. Gut Microbiome and Biomarkers for Unfavorable Microbiome

The human gut harbors a large population of microbes such as bacteria, viruses, protozoa, and fungi and has an estimated biomass of 1.5 kg, which is comparable in size to the liver, so it can be considered an organ. Therefore, any changes to this composition can lead to a diseased condition. Gut dynamics can be influenced by several factors, such as age, diet, gender, ethnicity, and environment [35,36]. A study by Brooks et al. showed how differences across ethnicities can potentially affect the microbial composition. In this comprehensive study, including 1673 individuals in the United States, the authors reported twelve microbial genera and families that reproducibly vary by ethnicity [35]. Diet and gut microbiome are the deciding factors of human health. Microbial ecology, genotype, and their proportion affect energy and nutrient harvesting. To understand these interrelations, a study model representing the human gut ecosystem was prepared by Turnbaugh et al. using germ-free C57BL/6J mice by transplanting fresh or frozen adult human fecal microbial communities. They observed altered microbiome gene expression, increased adiposity, and a changed representation of metabolic pathways in the microbiome after switching from a low-fat, plant polysaccharide-rich diet to a high-fat, high-sugar “Western” diet [37].

Disturbance in the microbiome can result in disease conditions, evidence and a discussion of which can be found in this review (Table 1). Dectin-1 is a pattern-recognition receptor and innate immune response modulator protein that potentially serves as a biomarker to identify people with a potentially unfavorable gut microbiome. Dectin-1 gene expression in subcutaneous adipose tissue was investigated in the context of obesity and associated inflammatory markers. The results showed a correlation between the expression of dectin-1 transcripts and various proinflammatory cytokines, chemokines, and their cognate receptors in terms of body mass index, fat percentage, and monocyte/macrophage markers (CD16, CD68, CD86, and CD163). These results suggest that dectin-1 can be used as an adipose tissue biomarker of metabolic inflammation in obesity [38].

Similarly, short-chain fatty acids (SCFAs) like propionate, butyrate, and acetate have been found to be another biomarker for multiple sclerosis pathogenesis. Upon comparing the serum SCFA levels of multiple sclerosis patients with those of healthy ones for immune cell abundance and phenotype as well as with other relevant serum factors, a significant reduction in propionate levels in the serum of the patients was noticed. There is a positive correlation between serum propionate and the frequencies of circulating T follicular regulatory cells and T follicular helper cells. However, butyrate levels are associated positively with the frequencies of IL-10-producing B-cells and negatively with the frequencies of class-switched memory B-cells. In addition, acetate levels are negatively correlated with TNF production by polyclonally activated B-cells. Therefore, serum SCFA levels can be associated with changes in circulating immune cells and biomarkers implicated in the development of multiple sclerosis [51]. Nevertheless, propionate and acetate produced by Propionibacterium during fermentation processes are major cytotoxic components secreted by the bacteria. SCFAs can kill human colorectal carcinoma cell lines in multiple ways, including apoptosis, a loss of mitochondrial transmembrane potential, the generation of reactive oxygen species, caspase-3 processing, influencing extracellular pH, nuclear chromatin condensation, and necrosis. These results suggest that SCFAs produced by propionibacteria could exhibit deleterious effects on cancerous cells and probiotic properties which are efficient in digestive cancer prophylaxis [52].

Due to the availability of advanced techniques like fluorescent oligonucleotide hybridization, it is now easier to figure out the microbiome composition by labeling bacterial cells, which can be easily and readily analyzed by flow cytometry [53] and molecular-phylogenetic methods [54]. The probes labeled with tetramethylrhodamine are used to complement short-sequence elements common to phylogenetically coherent assemblages of microorganisms within 16S rRNA and hybridize to suspensions of fixed cells. Similarly, molecular-phylogenetic methods also help to identify microbes by taking advantage of polymerase chain reaction amplification and phylogenetic analysis of small-subunit ribosomal RNA gene sequences. This technique also helps design sequence-based tools for identifying, tracking, and diagnosing the presence of microbes in complex samples [54].

The method of amplicon sequencing the bacterial 16S rRNA gene has been used to discriminate between pancreatic cancer patients and controls, where fourteen bacterial features were found to be altered [55]. Bacterial 16S rDNA Illumina^®^ sequencing and 16S rDNA quantitative polymerase chain reaction methods help determine the association between the urinary microbiome and prostate cancer. These methodologies were used in a study by Shrestha et al., 2018, to describe the association between the prevalence of pro-inflammatory bacteria and uropathogens in the urinary tract of men with prostate cancer, which is related to chronic inflammation [56]. A study in 2018 by Liss et al. showed that *Streptococcus* and *Bacteroides* species were higher in the rectal swab samples of men suffering from prostate cancer [26]. Similarly, another case–control pilot study on 20 men with prostate cancer showed a higher relative abundance of *Bacteroides massiliensis* in the gut microbiome as compared to the controls [50]. Patients undergoing treatment with androgen receptor axis-targeted therapy (ATT) showed a higher relative abundance of *Ruminococcaceae* spp. and *Akkermansia muciniphila*, which are involved in steroid hormone biosynthesis [57]. These studies indicated the importance of specific microbial compositions that can be utilized as a potential biomarker of cancer pathogenesis.

On the other hand, the association between the microbiome and the prognosis of head and neck squamous cell carcinoma (HNSCC) was uncertain. Since then, several studies have highlighted the importance of the microbiota in the development and prognosis of several types of cancer. A recent study on a retrospective cohort of 129 patients with oral cavity squamous cell carcinoma (OSCC) and oropharyngeal squamous cell carcinoma (OPSCC) reported greater abundances of the genera *Bacillus*, *Lactobacillus*, and *Sphingomonas* to be linked with favorable prognoses in head and neck squamous cell carcinoma (HNSCC) [58].

Cancer patients suffering from renal cell carcinoma (RCC) and non-small-cell lung cancer (NSCLC) treated with antiprogrammed cell death ligand-1 (PD-L1) or PD-L1 plus CTLA-4 mAb monotherapy who were also undergoing treatment with antibiotics (β-lactam or quinolones for pneumonia or urinary tract infections) in the beginning were examined for thirty days for gut microbiota diversity and composition leading to dysbiosis. In this study, the results suggested those patients’ receiving antibiotics suffered an increased risk of primary progressive disease, which means the antibiotics were affecting or lowering the effectiveness of immune-checkpoint inhibitor therapy. The antibiotic treatment increased the risk of primary progressive disease in both types of cancer patients, suggesting that antibiotics reduced the clinical benefits of immune-checkpoint inhibitor therapy in the RCC and NSCLC patients, which can be modulated by altering the gut microbiota composition [59]. This fact can be seen in a study where the ablation of the microbiome with immunogenic reprogramming protected against preinvasive and invasive pancreatic ductal adenocarcinoma (PDA). The removal of the microbiome resulted in a reduction in myeloid-derived suppressor cells and increased M1 macrophage and T cell differentiation and activation. This strategy enabled efficacy for checkpoint-targeted immunotherapy by upregulating PD-1 expression via activating Toll-like receptors in monocytic cells [60].

Anticancer drugs alter the intestinal microbiota in such a way they cause an induction of immune responses; for example, cyclophosphamide imposes antitumor immune responses by altering the composition of the small intestine microbiota and induces the translocation of selected species of Gram-positive bacteria (*Lactobacillus johnsonii*, *Lactobacillus murinus*, and *Enterococcus hirae*) into secondary lymphoid organs in mouse model. These bacteria help induce the generation of pathogen-specific T helper 17 (Th17) cells and memory immune responses. This fact was demonstrated by Viaud et al., where the authors showed how tumor-bearing mice treated with antibiotics got rid of Gram-positive bacteria, causing a reduction in the pathogen-specific Th17 responses that made them resistant to cyclophosphamide. Seven days postcyclophosphamide treatment, there was a significant reduction in the frequencies of CD103^+^CD11b^+^ dendritic cells and TCRαβ^+^CD3^+^ T cells that were expressing the transcription factor RORγt in the lamina propria (LP) of the small intestine. However, the adoptive transfer of pathogen-specific Th17 cells to vancomycin-treated mice partially restored the antitumor efficacy of cyclophosphamide, suggesting the efficacity of the gut microbiome in strengthening anticancer immune responses [61].

An intact gut microbiome is required to obtain favorable patient outcomes during cancer chemotherapies because microbiota have a direct influence on local and systemic inflammation and influence disease progression and treatment. It has been reported that disruption to the microbiota impairs the response of subcutaneous tumors to CpG-oligonucleotide immunotherapy and platinum chemotherapy. This aspect can be seen in detail in a study by Iida et al., where C57Bl/6 mice were pretreated with an antibiotic cocktail containing vancomycin, imipenem, and neomycin before tumor inoculation, and this continued till the end of the study. The treatment of the antibiotic mix reduced the frequency of monocyte-derived Ly6C^+^ MHC class II^+^ cells and Ly6G^high^ neutrophils. The authors observed that the production of cytokines and tumor necrosis after the CpG-oligonucleotide treatment was lower in the antibiotic-treated or germ-free mice, suggesting that the optimal response to chemotherapy demands an effective and intact gut microbiome [62]. In a study, T cell infiltration into solid tumors facilitated immunotherapy. Immune cell infiltration into the tumor site makes immunotherapies work, as described by Sivan et al. by using a 16S ribosomal RNA sequencing method to identify functionally relevant bacterial taxa. A comparative analysis between quick-growing-tumor-bearing mice from Jackson Laboratory and slow-growing-tumor-bearing mice from Taconic Farms revealed that 257 taxa were significantly different. A further analysis identified *Bifidobacterium*’s association with antitumor effects since its combined treatment with PD-L1-specific antibody therapy abolished tumor outgrowth. This resulted in the accumulation of antigen-specific T cells and induced IFN-γ by enhancing dendritic cell function, leading to enhanced CD8^+^ T cell priming and accumulation in the tumor microenvironment [63].

Research has shown how disrupting β-glucuronidase enzyme activity, which is present in the commensal microbiota, can alleviate toxicity caused by anticancer drugs. There was a study focused not on altering the bacterial population but on targeting symbiotic bacterial β-glucuronidases, which cause severe diarrhea upon colon cancer chemotherapeutic CPT-11 (irinotecan) treatment. CPT-11 is a derivative of a known potent antineoplastic compound, Camptothecin, which is undergoing clinical use but still elicits pronounced side effects that limit its efficacy. The authors screened bacterial β-glucuronidase inhibitors by high-throughput screening using a β-glucuronidase assay. This investigation selected potential inhibitors that had no effect on orthologous mammalian enzymes since crystallographic studies revealed that the *E. coli* enzyme contains a 17-residue “bacterial loop”, which is not present in the human ortholog, which makes it selective to bacterial enzymes but not to mammalian enzymes. The findings were confirmed in mice, showing that the oral administration of a β-glucuronidase inhibitor protected Balb/cJ mice from CPT-11-induced toxicity without losing the glandular structures of the intestinal tissues and keeping the gastrointestinal epithelium intact, thereby reducing both diarrhea and bloody diarrhea. Therefore, drugs should be designed to inhibit undesirable enzyme activities rather than alter the actual microbiome to enhance chemotherapeutic efficacy [64].

## 4. Probiotics Improve Cancer Treatment

There are diverse procedures these days to go with surgery, such as chemotherapy, radiotherapy, immunotherapy, and hormonal therapy, which produce a lot of side effects, especially abdominal pain, severe gastric issues, constipations, nausea, vomiting, diarrhea, taste disturbances, mucositis, and swallowing difficulties, thus affecting the quality of life of these patients [1,65]. Thanks to advanced research and therapeutic strategies that started using probiotics to modify the microbiota to manage these complications (Table 2), a number of studies have proposed and confirmed that probiotics can be effective at controlling the growth of cancer cells [1,66,67,68,69].

The intake of fermented dairy products reduces the risk of cancer by inducing immune responses and creating balanced and healthy gut microbiomes to support all therapies. A detailed, 12-year-long study conducted to determine the effect of yogurt consumption on cancer prevention in volunteers of the EPIC-Italy cohort suggested the protective role of yogurt against colorectal cancer (CRC). This finding suggests that yogurt should be part of a diet to prevent the disease [72].

The exopolysaccharides derived from probiotic yeast *Kluyveromyces marxianus* and *Pichia kudriavzevii* have shown inhibitory effects on different colon cancer cell lines, hindering the AKT-1, mTOR, and JAK-1 pathways and induced apoptosis [97]. Patients receiving CRC and undergoing a perioperative probiotic administration of combined probiotics containing *Bifidobacterium longum* (≥1.0 × 10^7^ cfu/g), *Lactobacillus acidophilus* (≥1.0 × 10^7^ cfu/g), and *Enterococcus* faecalis (≥1.0 × 10^7^ cfu/g) showed that this significantly influenced the recovery of bowel function [98].

One of the most widely used probiotic strains is Lactobacillus, lactic-acid-producing bacteria that is continuously studied and used to improve the function of the gut system. In this context, a study evaluated *Lactobacillus reuteri* FLRE5K1 for its antimelanoma activity in cell assays and animal models. The results showed that treatment with *Lactobacillus reuteri* FLRE5K1 reduced the incidence of tumors as compared to the model group. FLRE5K1 did not inhibit melanoma tumor mass but prolonged the survival of tumor-bearing mice. The uptake of *Lactobacillus reuteri* FLRE5K1 by immune cells induced elevated levels of TNF-α and INF-γ in serum, suggesting that it stimulated immune responses and limited the infiltration of melanoma cells with blood circulation [99]. In addition to prolonging the survival of tumor-bearing mice, *Lactobacillus reuteri* helps alleviate liver injury and intestinal inflammation induced by D-galactosamine in Sprague–Dawley (SD) rats. Probiotics work by maintaining redox homeostasis by increasing the expression of the intestinal tight junction protein proteins ZO-1 and occludin, which help the intestinal barrier morphology to remain intact. The supplementation of *Lactobacillus reuteri* inhibited intestinal apoptosis by downregulating the expression of caspase-3, activating Nrf-2 nuclear translocation, and elevating the induction of HO-1 via activating phosphoinositide 3-kinase/protein kinase B (PI3K/Akt), protein kinase C (PKC), and their phosphorylated forms [100]. Treatment with anticytotoxic T-lymphocyte-associated protein 4 (CTLA-4) or PD-1/PD-L1 triggers severe inflammatory side effects such as colitis due to changes in the composition of the gut microbiota, especially a reduction in the abundance of Lactobacillus. In this case, the oral administration of *Lactobacillus reuteri* inhibited the development and progression of colitis by restoring the colon structure and reducing leukocyte infiltration. This treatment decreased the levels of inflammatory cytokines (KC, TNF-α, IFN-γ, and IL-6) and reversed the loss of body weight and inflammatory status induced by antiCTLA-4 or antiPD-L1 immunotherapy [101].

Another example, *Lactobacillus rhamnosus* GG, showed protective benefits against colon cancer by suppressing proliferation and promoting apoptosis in colon cancer cell lines [66]. Consequently, the administration of a probiotic combination could indeed restore immune function and the composition of an altered gut microbiota. The consumption of yogurt containing high counts of viable *Streptoccocus thermophilus* and *Lactobacillus delbrueckii* subsp. *bulgaricus* was inversely associated with the risk of colorectal cancer by lowering the levels of cancer biomarkers, stimulating anticancer effects, and increasing the survival rate [72]. Similarly, a double-blind trial conducted with 138 patients suffering from superficial transitional cell carcinoma of the bladder showed the prophylactic effects of consuming *Lactobacillus casei* in preparation for treatment [102]. *Lactobacillus casei* BL23 treatment in C57BL6 mice protected against colitis-associated colorectal cancer by inducing immunomodulatory effects through downregulating IL-22 cytokines and antiproliferative effects through upregulating *caspase-7*, *caspase-9*, and *Bik* RNA expression and downregulating Ki67 levels [82]. Nonetheless, this strain induces IL-17A, a pro-inflammatory cytokine produced by TH17 cells that participates in antitumor effects. In this study, Jacoutan et al. developed a recombinant strain of *Lactobacillus casei*, BL23, capable of secreting IL17A to understand the involvement of IL17A in cancer. Their results showed that in around 26% of the mice treated intranasally with *L. lactis*-IL-17A and challenged with a tumor cell line, TC-1 cells derived from lung epithelial cells of C57BL/6 mice remained protective against the tumors, confirming the antitumor effect of IL17 cytokines through inducing IL-6 secretion in splenocytes [89]. Another population-based case–control study based on a questionnaire and interview was conducted to assess the combination effects of the regular consumption of *Lactobacillus casei* Shirota (BLS) and isoflavones on the incidence of breast cancer in Japanese women, in which 306 cases with breast cancer and 662 controls aged 40–55 were included in the analyses. Their analysis suggested the inverse association between soy consumption and breast cancer incidence could be due to isoflavones’ anticancer properties and their inducing natural killer cell activity [76].

Moreover, *Streptococcus thermophilus* is a lactic acid bacterium known to be used as a starter culture for a number of dairy products to produce folate during growth. Its cytotoxic effect on cancer cells was tested in a study using eight *S. thermophilus* strains (1F8CT, MTH17CL396, M17PTZA496, TH982, TH985, TH1435, TH1436, TH1477) isolated from curd, cheese, whey, and raw milk dairy products in Italy [90]. This group evaluated all the strains for their number of health-related traits, such as the production of folic acid and anticancer properties, by evaluating their ability to attach and inhibit the growth of human HT-29 colorectal adenocarcinoma cells. The results revealed that two strains of *S. thermophilus*, namely M17PTZA496 and TH982, have probiotic properties along with anticancer activity and folate production capability. These strains were superior to *Lactobacillus rhamnosus* GG for some of these properties, which suggests that they can pose better health benefits if used commercially, but before that, they need to be studied in more detail.

In addition to probiotics, prebiotics can also manage the gut microbiota, alleviate the side effects of cancer therapies, and prevent gastric cancer. Prebiotics promote the growth of beneficial bacteria and possess protective effects against colon carcinogenesis. The fermentation of prebiotics by gut microflora generates short-chain fatty acids that alter the gene expression and differentiation of tumor cells [103]. An in vivo study on azoxymethane/dextran–sodium–sulfate-induced colorectal cancer in C57BL/6 mice has shown prebiotics’ preventive effects against colorectal cancer [104]. A clinical trial among colorectal cancer patients showed that preoperative oral supplementation can alleviate postoperative complications. Patients received a daily oral intake of 30 g of prebiotic supplements, which showed positive effects on immune status [105]. Not only does the intake of prebiotics prevent cancer, but it also increases the prevalence of commensal microbiota (*Bacteroides*, *Bifidobacterium*, *Escherichia-Shigella*, and *Enterococcus*) in these individuals. Another randomized double-blind trial conducted on 73 colorectal cancer patients with preceding colorectal operations found that patients treated with an oral intake of higher concentrations of lactic acid bacteria did not have severe inflammatory responses after surgery [106].

The gut microbiome impacts the therapeutic outcomes of checkpoint blockade immunotherapy in human cancer patients. An examination of the oral and gut microbiome of melanoma patients under the treatment of antiPD-1 immunotherapy demonstrated a significant difference in the diversity and composition of the gut microbiome. Patients whose fecal samples showed a high diversity and abundance of *Ruminococcaceae/Faecalibacterium* had significantly better progression-free survival. These changes in the gut microbiome may modulate therapy responses to antiPD-1 immunotherapy in melanoma patients by modulating immune responses. Patients with a higher abundance of these strains had higher levels of CD4+ and CD8+ T cells in their systemic circulation [86]. In line with this, T cell responses during immunotherapies depend on distinct Bacteroides species, such as *Bacteroides thetaiotaomicron* or *Bacteroides fragilis.* The CTLA blockade effect on tumors was not enough in the mice treated with antibiotics or the germ-free mice, while this defect was overcome by the adoptive transfer of *B. fragilis*-specific T cells [107]. A study applied a combination of eleven bacterial strains (*Parabacteroides distasonis*, *Parabacteroides gordonii*, *Parabacteroides johnsonii*, *Alistipes senegalensis JC50*, *Paraprevotella xylaniphila*, *Bacteroides uniformis*, *Bacteroides vulgatus*, *Eubacterium limosum*, *Ruthenibacterium lactatiformans strain 585-1*, *Phascolarctobacterium succinatutens YIT 12067*, *Fusobacterium ulceranus*) from healthy human donor feces to syngeneic tumor mouse models. All the strains altogether mediated local and systemic immunomodulation by enhancing the recruitment of IFNγ^+^ CD8 T cells in the intestine without causing inflammation due to CD103^+^ dendritic cells and major histocompatibility (MHC) class Ia molecules. This strain mixture not only enhanced host resistance against *Listeria monocytogenes* infection but also improved anticancer immunity in the tumor models [108]. Similarly, fecal transplantation therapy by low-volume enema helped to overcome side effects from chronic antibiotic (oral vancomycin) therapy [109]. Treatment with antibiotics ameliorates the benefits of immune checkpoint inhibitors. The administration of fecal microbiota from cancer patients who responded to immune checkpoint inhibitors helped to reverse gut microbiota dysbiosis to a healthy gut environment in mouse models. These patients’ stool was highly abundant in *Akkermansia muciniphila*; therefore, its oral supplementation restored the efficacy of the PD-1 blockade. The adjuvant effect of *A. muciniphila* induced dendritic cells to secrete IL-12, a Th1 cytokine with importance in the immunogenicity of the PD-1 blockade in eubiotic conditions, which helped recruit CCR9+CXCR3+CD4+ T lymphocytes into mouse tumor beds within 48 h after the first injection in mesenteric lymph nodes [87].

*Enterococcus faecium* RM11 and *Lactobacillus fermentum* RM28 isolated from fermented dairy milks trigger the antiproliferation of colon cancer cells at rates of 21–29% and 22–29%, respectively [71]. Colorectal cancer patients treated for six months with probiotics containing a 30 × 10^9^ colony-forming unit mixture of six *Lactobacillus* and *Bifidobacteria* strains showed a significant reduction in the level of pro-inflammatory cytokines, TNF-α, IL-6, IL-10, IL-12, IL-17A, IL-17C, and IL-22, and also avoided the requirement for antibiotics and showed no infections [92].

Collectively, all these studies suggest that any change or dysbiosis in the gut microbiota has a significant effect on cancer growth, development, and treatment [110,111]. The challenges faced during cancer therapies, i.e., chemotherapy, surgery, immunotherapy, and radiotherapy, which mainly include diarrhea, mucositis, abdominal pain, and reduced quality of life, or the adverse effects caused by heavy antibiotic treatment can be alleviated by encouraging alternative therapeutic methods along with cancer therapies involving adjuvant therapies like the application of probiotics in clinical management. Including probiotics, synbiotics, or probiotics in a correct manner can help to resolve these cons (Table 3). The administration of yeast probiotics has been shown to have an adjuvant property in treating colorectal cancer by several means, including boosting immunological and antioxidant properties [112]. Antioxidant properties have greater implications for cancer treatment, as can be seen in the study where *Lactobacillus plantarum* AS1 (AS1) showed its direct impact on colon cancer treatment by inducing activities of antioxidant enzymes, e.g., lipid peroxide, superoxide dismutase, catalase, glutathione-S transferase, alkaline phosphatase, and acid phosphatase, in the colon and plasma of cancer-bearing animals [73].

## 5. Conclusions

Taken together, this article highlights the quality and type of food that should be consumed and the types of food that can enhance the risk of cancer. Disturbance in the gut microbiome leads to resistance to cancer therapies and can ameliorate their beneficial effects. The gut microbiota can be modified to benefit immune responses and support immunotherapies by using probiotics and prebiotics as adjuvants. Several clinical trials and in vivo and in vitro experiments have proposed the use of these adjuvant therapies, as they can shape the intestinal microbiota, which is disturbed in diseased conditions, and help to promote overall wellness. In this review, we focused on the identification of the microbiome as a biomarker of diseased conditions and how the specific usage of probiotics, or some strains, can be useful to improve the efficacy of treatments and eliminate their side effects.

## Figures and Tables

**Table 1:** Prevalence of specific microbiota for different cancer types.

Biomarkers	Cancer Type	Abundance	Ref.
*Veillonella*, *Fusobacterium*, *Prevotella*, *Porphyromonas*, *Actinomyces* and *Clostridium*, *Haemophilus*, *Enterobacteriaceae* and *Streptococcus* spp. and *Candida albicans*	Oral carcinoma	Elevated in tumor sites	[39]
*Capnocytophaga gingivalis*, *Prevotella melaninogenica* and *Streptococcus mitis*	Oral squamous cell carcinoma (OSCC)	Elevated in the saliva of individuals with OSCC	[40]
*Fusobacterium nucleatum*, *S salivarius: Streptococcus vestibularis*, *Prevotella oris*, and *Rothia mucilaginosa*	Head and neck squamous cell carcinoma (HNSCC)	HNSCC patients had a significant loss in richness and diversity of microbiota species	[41]
*Fusobacterium nucleatum*	HNSCC	Elevated in the saliva	[42]
*Corynebacterium and Kingella*	HNSCC	Greater oral abundance of these commensals is associated with decreased risk of HNSCC	[43]
*Capnocytophaga*, *Pseudomonas*, and *Atopobium*	OSCC	Highly abundant in biopsy tissue	[44]
*Lautropia*, *Staphylococcus*, and *Propionibacterium*	Fibroepithelial polyp	Highly abundant in biopsy tissue	[44]
*Treponema denticola*, *Streptococcus mitis*, and *Streptococcus anginosus*	Esophageal cancer	Induction of inflammatory cytokines	[45]
*Group G streptococci*	Colon cancer	Large amount of pericardial effusion	[46]
*Fusobacterium nucleatum*	Colorectal carcinoma	Over-representation in tumor specimen	[47]
*Neisseria elongate*, *Streptococcus mitis* and *Granulicatella adiacen*	Pancreatic cancer and chronic pancreatitis	Salivary microbiota as an informative source for discovering noninvasive biomarkers of systemic diseases	[48]
*Helicobacter pylori*	Dysplasia or gastric cancer	Presence of *H. pylori* at baseline was associated with an increased risk of progression to dysplasia or gastric cancer	[49]
*Streptococcus* and *Bacteroides species (Bacteroides massiliensis)*	Prostate cancer	Higher relative abundance in prostate cancer cases compared to controls.	[26,50]

**Table 2:** Probiotics to support cancer treatment.

Probiotics	Cancer Type	Study Model	Effect	Ref.
*Propionibacterium freuden reichii*	Colorectal cancer	HT-29 cells	Induced cell cycle arrest in the G2/M phase	[70]
*Enterococcus faecium* RM11, *Lactobacillus fermentum* RM28	Colon cancer	Caco-2 cells	Triggered antiproliferation of colon cancer cells	[71]
Yogurt probiotics	Colorectal cancer	Clinical trial		[72]
*Lactobacillus plantarum AS1*	Colon cancer	Rat	Antioxidant-dependent mechanism	[73]
*Lactobacillus casei* and *Lactobacillus rhamnosus* GG	Colorectal cancer	HCT-116 cells	Decreased metalloproteinase-9 activity and increased levels of tight junction protein zona occludens-1	[74]
*Lactobacillus plantarum* AS1	Colorectal cancer	Male Wistar rats	Antioxidant property reduced tumor volume diameter and total number of tumors	[73]
*Lactobacillus acidophilus*	Breast cancer	Balb/C inbred female mice	Induces production of IFNγ, IL-4, and TGF-β	[75]
*L. casei* Shirota	Breast cancer	Case –control study	NK cell activation and NK cell-mediated antitumor activity	[76]
*Lactobacillus casei* Shirota	Breast cancer	Population-based case –control study	Enhanced NK cell activity-mediated antitumor activity	[76]
*Lactobacillus fermentum*	Colorectal cancer	Caco-2 colon cancer cell	Antiproliferative activity	[77]
Dead nano-sized *Lactobacillus plantarum*	Colon cancer	Balb/c mice	Suppressed inflammation, induced cell cycle arrest and apoptosis, and enhanced IgA secretion	[78]
*Lactobacillus lactis* NK34	Lung, colon, gastric adenocarcinoma, breast cancer	SK-MES-1, DLD-1, HT-29, LoVo, AGS, MCF-7, and RAW 264.7 cells	Reduced production of nitric oxide and proinflammatory cytokines (tumor necrosis factor-α, interleukin-18, and cyclooxygenase-2)	[79]
*Lactobacillus casei* ATCC334	Colon cancer	Human colon cancer cell lines (Caco2_bbe_, SKCO-1, and SW620) and Xenografts (SW620 cells injected into male BALB/c nude mice)	Ferrichrome-induced apoptosis by activating C-jun N-terminal kinase and suppressed tumor growth	[80]
*Lactobacillus casei* ATCC 393	Colon cancer	Murine (CT26) and human (HT29) colon carcinoma cell lines	Apoptotic cell death and upregulation of TRAIL in colon carcinoma cells	[81]
*Lactobacillus casei* BL23	Colitis-associated colorectal cancer	C57BL6 mice	Immunomodulatory effect, mediated through the downregulation of the IL-22 cytokine, and an antiproliferative effect, mediated through the upregulation of caspase-7, caspase-9, and Bik	[82]
*Lactobacillus acidophilus* ATCC 314 and *Lactobacillus fermentum* NCIMB 5221	Colorectal cancer	Apc ^Min/+^ CRC mouse	Downregulated proliferation markers (Ki-67, E-cadherin, β-catenin)	[83]
*Lactobacillus reuteri* NCIMB 701,359	Colorectal cancer	DLD-1 cell line	Probiotic-derived protein, p8, inhibits p53-p21-Cyclin B1/Cdk1 signal pathway	[84]
*Acetobacter syzygii*	Squamous cell carcinoma	Human oral cancer (KB) and human normal epithelial (KDR) cell lines	Induced apoptosis	[85]
*Bifidobacterium longum*, *Collinsella aerofaciens*, and *Enterococcus faecium*	Metastatic melanoma	Melanoma patients	Enhanced systemic and antitumor immune responses mediated by increased antigen presentation and improved effector T cell function	[86]
*Akkermansia muciniphila*	Non-small-cell lung cancer, renal cell cancer, and urothelial cancer	Mice	Increasing the recruitment of CCR9+CXCR3+CD4+ T lymphocytes	[87]
*Lactobacillus acidophilus* 20079	Colon cancer	Colon cancer (CaCo-2) and human breast cancer (MCF7) cell lines	Increased apoptosis in sub-G0/G1 cell cycle phase, stimulated immune response, and inactivated NF-κB inflammatory pathway	[88]
Recombinant *Lactococcus lactis*		Mouse allograft model of human papilloma virus (HPV)-induced cancer and TC-1 cell line	Secreting IL-17 to stimulate the TH17 pathway	[89]
*Streptococcus thermophilus*	Colorectal cancer	HT-29 human colorectal adenocarcinoma cells	High production of folic acid, tyramine, and histamine; high cytotoxic to cancer cells	[90]
*Bifidobacterium breve* lw01	Head and neck cancer	SCC15, CAL 27, and WSU-HN6 cell lines	Increased expression of cell apoptosis protein caspase 3 and PARP and increased proportion of Cl-PARP/PARP	[91]
*Lactobacillus* and *Bifidobacteria* strain	CRC	Randomized double-blind placebo-controlled trial	Interfered with the signaling pathways to stimulate or suppress the level of cytokine production	[92]
*Enterococcus faecalis*	Colorectal cancer	C57BL/6 mice	Inhibited NLRP3 inflammasome activation in macrophages	[93]
*Lactobacillus delbrueckii* ssp. *bulgaricus B3*	Colon cancer	HT-29 cells	Inhibited cell proliferation in HT-29 via apoptosis	[94]
Recombinant *Lactococcus lactis*	Colorectal cancer	Murine fibroblast 3T3 L1 cell lines and mouse allograft model of human papilloma virus-induced cancer	Efficiently secretes biologically active IL-17A cytokines	[89]
*Lactobacilli cocktail*	Colorectal cancer	HT-29 colon carcinoma cells	Antitumor effects on HT-29 cells by modulating the Notch and Wnt/β-catenin pathways	[95]
*Lactobacillus reuteri*	Colon Cancer	Colon cancer stem-like cells (HT29-ShE)	Antimetastatic and antiproliferative	[96]
*Kluyveromyces marxianus* and *Pichia kudriavzevii*	Colon cancer	Colon cancer cell lines (SW-480, HT-29, HCT-116)	Hindered AKT-1, mTOR, and JAK-1 pathways and induced apoptosis	[97]

**Table 3:** Clinical trial using probiotics during cancer treatment.

Probiotics	Cancer Type (Year)	Effect and Results	Ref.
*Lactobacillus casei LC9018*	Lung cancer (malignant pleural effusions secondary to lung cancer) (1991)	Significantly greater improvement in performance status and symptoms (chest pain, chest discomfort, and anorexia). Response rate for treatment with intrapleural doxorubicin plus LC9018 was significantly higher.	[113]
*Lactobacillus rhamnosus GG ATCC 53103*	Colorectal cancer (lactose intolerance associated with adjuvant 5-fluorouracil-based chemotherapy) (2007)	Bowel mucosal injury associated with 5-fluorouracil (5-FU) treatment. The frequency of severe 5-FU-based chemotherapy-related diarrhea was reduced.	[114]
*Lactobacillus casei DN-114 001*	Endometrial adenocarcinoma patients (2008)	Oral supplementation did not reduce the incidence of radiation-induced diarrhea. Showed a significant effect on stool consistency.	[115]
*Lactobacillus acidophilus* plus *Bifidobacterium bifidum*	Cervical cancer (2010)	Reduced the incidence of radiation-induced diarrhea and the need for antidiarrheal medication. Had a significant benefit on stool consistency.	[116]
*Lactobacillus rhamnosus GG*	Colorectal cancer (2013)	Lactobacillus intervention during 5-fluorouracil chemotherapy. Reduced diarrhea only in nonproducers of methane.	[117]
*Lactobacillus acidophilus*	Prostate cancer (2013)	Useful in reducing the percentage volume change of the rectum, a crucial factor in prostate movement.	[118]
*Bifidobacterium*, *Lactobacillus* and *Streptococcus thermophilus*	Acute radiation enteritis (2014)	Treated group showed better food intake than control group.	[119]
*Lactobacillus acidophilus*, *Lactobacillus rhamnosus*, *Lactobacillus casei DN-114001* and *Bifidobacterium bifidum*	Abdominal or pelvic cancer (2014)	Beneficial effects on treatment of radiation-induced diarrhea. Improved the quality of patients’ lives.	[120]
*Lactobacillus acidophilus BMC12130*, *Lactobacillus casei BCMC12313*, *Lactobacillus lactis BCMC12451*, *Bifidobacterium bifidum BCMC02290*, *Bifidobacterium longum BCMC02120* and *Bifidobacterium infantis BCMC02129*	Colorectal cancer (2017)	Improved quality of life. Reduced certain inflammatory biomarkers (IL-6) and relieved certain side effects of chemotherapy.	[121]
*Bifidobacterium breve strain Yakult*, *Lactobacillus casei strain Shirota*	Esophageal cancer (2017)	Reduced the occurrence of adverse events from chemotherapy through adjustments to the intestinal microbiota. Concentrations of acetic acid and propionic acid were significantly higher in the synbiotics group. The frequencies of severe lymphopenia and diarrhea were significantly less. Febrile neutropenia occurred less in the synbiotics group.	[122]
*Lactobacillus acidophilus LA-5* plus *Bifidobacterium animalis* subsp. *lactis BB-12*	Cervical cancer (2019)	Incidence of diarrhea was reduced. Severity of abdominal pain was significantly reduced.	[123]
*Lactobacillus brevis CD2*	Head and neck cancer (2019)	Modulated homeostasis of the salivary microbiota.	[124]
*Bifidobacterium longum*, *Lactobacillus lactis* and *Enterococcus faecium*	Nasopharyngeal carcinoma (2019)	Significantly increased the immune responses. Reduced the severity of oropharyngeal mucositis.	[125]
*Lactobacillus acidophilus*, *Lactobacillus casei*, *Lactobacillus plantarum*, *Lactobacillus rhamnosus*, *Bifidobacterium lactis*, *Bifidobacterium bifidum*, *Bifidobacterium breve*, *Streptococcus thermophilus*	Colorectal cancer (2019)	Significant reduction in postoperative complications in the localization of tumors on the rectum −33.3% and the ascending colon −16.7%.	[126]
*Streptococcus salivarius M18*	Head and neck cancer (2020)	Improvement in periodontal screening and plaque index scores was observed.	[127]
*Lactobacillus acidophilus*, *Lactobacillus rhamnosus*, *Bifidobacterium longum* and *Saccharomyces boulardii*	Head and neck cancer (2020)	Significant reduction in *Candida* spp. Counts, specifically *Candida glabrata* and *Candida tropicalis*.	[128]
*Lactobacillus plantarum MH-301 (CGMCC NO. 18618)*, *L. rhamnosus LGG-18 (CGMCC NO. 14007)*, *Lactobacillus acidophilus* and *Bifidobacterium animalis* subsp. *lactis LPL-RH (CGMCC NO. 4599)*	Gastric cancer (2021)	Probiotic compounds can restore gut microbiota homeostasis, reduce inflammation, maintain intestinal mucosal barrier and immunity, and finally promote recovery after gastrectomy.	[129]
*Bifidobacterium longum*, *Lactobacillus acidophilus* and *Enterococcus faecalis*	Breast cancer (2022)	Probiotic supplement attenuates chemotherapy-related cognitive impairment in patients.	[130]
*Bifidobacterium infantis*, *Lactobacillus acidophilus*, *Enterococcus faecalis*, and *Bacillus cereus*	Thyroid cancer (2022)	Probiotics alleviated lack of energy, constipation, weight gain, and dry mouth and decreased the levels of fecal/serum LPS and plasma lipid indicators (total cholesterol, triglycerides, low-density lipoproteins, and apolipoprotein A).	[131]
*Lacticaseibacillus paracasei strain Shirota (YIT9029)*, *Bifidobacterium breve strain Yakult*,	Esophageal cancer (2022)	Incidences of grade 4 neutropenia and grades 2–4 diarrhea were significantly reduced.	[132]
*Bifidobacterium infantis*, *L. acidophilus*, *Enterococcus faecalis*, *and Bacillus cereus*	Colorectal cancer (2023)	Reduced chemotherapy-induced gastrointestinal complications, especially in the case of diarrhea. Probiotics also promoted the production of short-chain fatty acids, particularly increasing acetate, butyrate, and propionate.	[133]
